# Neuro-Ophthalmic Manifestations of Carotid Cavernous Fistulas: A Systematic Review and Meta-Analysis

**DOI:** 10.7759/cureus.65821

**Published:** 2024-07-31

**Authors:** Ali Al-shalchy, Ahmed S Al-Wassiti, Mohammed A Hashim, Younus M Al-Khazaali, Sura H Talib, Ali A Bani-Saad, Rania H Al-Taie, Mustafa Ismail

**Affiliations:** 1 Department of Surgery, College of Medicine, University of Baghdad, Baghdad, IRQ; 2 Department of Surgery, College of Medicine, Al-Nahrain University, Baghdad, IRQ; 3 Department of Surgery, College of Medicine, Al-Mustansiriya University, Baghdad, IRQ

**Keywords:** optic nerve, cranial nerve pathology, venous flow dynamics, neuro-ophthalmic presentations, carotid cavernous fistula

## Abstract

Carotid-cavernous fistulas (CCFs) are pathologic, arteriovenous communications between the carotid artery and cavernous sinus. They cause various complex neuro-ophthalmic symptoms by shunting the flow of arterial blood into the venous system. In this study, a systematic review is conducted on the neuro-ophthalmic presentations associated with CCFs. The Preferred Reporting Items for Systematic Reviews and Meta-Analyses 2020 guidelines were followed during the systematic review. We searched PubMed, Scopus, and Web of Science from inception to December 31, 2023. Articles written in English on patients with confirmed CCFs reporting clinical features, diagnostic modalities, treatment approaches, and outcomes were included. Abstracted data included demography, clinical presentations, venous flow dynamics, trauma history, investigative methodology, approaches to treatment, and outcomes. Overall, 33 studies with a total number of 403 patients were included. The mean age at presentation was 42.99 years for patients with direct CCFs and 55.88 years for those with indirect CCFs. Preponderance was observed in male patients with direct CCFs, constituting 51.56%, while females predominated in those with indirect CCFs, at 56.44%. The clinical symptoms in all patients with CCFs were proptosis in 58 cases (14.39%), conjunctival congestion in 29 patients (7.20%), diplopia in nine patients (2.23%), vision blurring in four patients (0.99%), eyelid swelling in five patients (1.24%), pain in the eye in three patients (0.74%), and an upper lid mass in one patient (0.25%). Endovascular treatments, including coil and Onyx embolization, have been effective in relieving clinical symptoms and arresting the progression of these symptoms. In conclusion, the common clinical features in CCFs usually underline proptosis, congestion, and diplopia, necessitating a comprehensive neuro-ophthalmological review. Prompt identification of the symptoms of blurred vision is crucial to avoid permanent damage. Lid swelling, ocular pain, and an upper lid mass are less common but equally essential presentations for comprehensive evaluation. The recognition of these variable presentations is essential not only for timely intervention but also for the improvement in patient outcomes, thus emphasizing the role of clinician awareness in managing CCF cases.

## Introduction and background

Carotid cavernous fistulas (CCFs) are abnormal connections that form between the carotid artery and the cavernous sinus. This anomaly allows arterial blood to flow directly into the venous system. The high rate of abnormal blood flow through the fistula can result in neuro-ophthalmic symptoms due to increased venous pressure and subsequent ocular and orbital congestion. Patients may experience proptosis, chemosis, diplopia, and other visual disturbances as a consequence [1,2].

CCFs can be classified into two main types: direct and indirect. Direct CCFs typically present as high-flow fistulas, often resulting from trauma or the spontaneous rupture of intra-cavernous aneurysms. This form involves a direct connection between the internal carotid artery and the cavernous sinus. On the other hand, indirect CCFs, also known as dural arteriovenous fistulas, are generally low-flow and tend to occur spontaneously. They are frequently associated with underlying conditions such as hypertension, atherosclerosis, and connective tissue disorders [3,4].

The neuro-ophthalmic symptoms of CCFs can vary widely depending on the type and size of the fistula, the direction of venous drainage, and any underlying conditions. These symptoms can be severe, often providing crucial diagnostic clues. Patients may exhibit ocular and cranial nerve abnormalities, such as proptosis, chemosis, and cranial nerve palsies that affect ocular motility. The increased venous pressure caused by abnormal venous drainage can lead to characteristic signs like ocular redness, reduced visual acuity, and pulsatile exophthalmos. Additionally, involvement of the third, fourth, fifth, and sixth cranial nerves, either directly or due to proximity to the cavernous sinus, can result in diplopia, ptosis, and facial pain or numbness. The varied presentation of these symptoms necessitates a high level of suspicion and thorough clinical evaluation [5].

The diagnosis of CCFs relies heavily on imaging techniques, with digital subtraction angiography (DSA) being the gold standard. Non-invasive methods such as computed tomography (CT), magnetic resonance imaging (MRI), and magnetic resonance angiography (MRA) are also useful for initial assessment and ongoing follow-up of patients [6,7].

Treatment options for CCFs include observation, neurosurgical interventions, and endovascular therapies. Endovascular treatment is often preferred due to its minimally invasive nature and high success rate. Procedures like trans-arterial and transvenous coil embolization, Onyx injection, and the use of other embolic agents have demonstrated good clinical outcomes in occluding the fistula and alleviating symptoms [8,9].

This systematic review aims to compile and summarize the current knowledge on the clinical features, diagnostic strategies, treatment modalities, and outcomes for patients with CCFs. Our goal is to provide a comprehensive review of evidence from various studies, offering a holistic view of managing this complex condition and identifying potential areas for future research.

## Review

Methods

Literature Search

**Figure 1:**
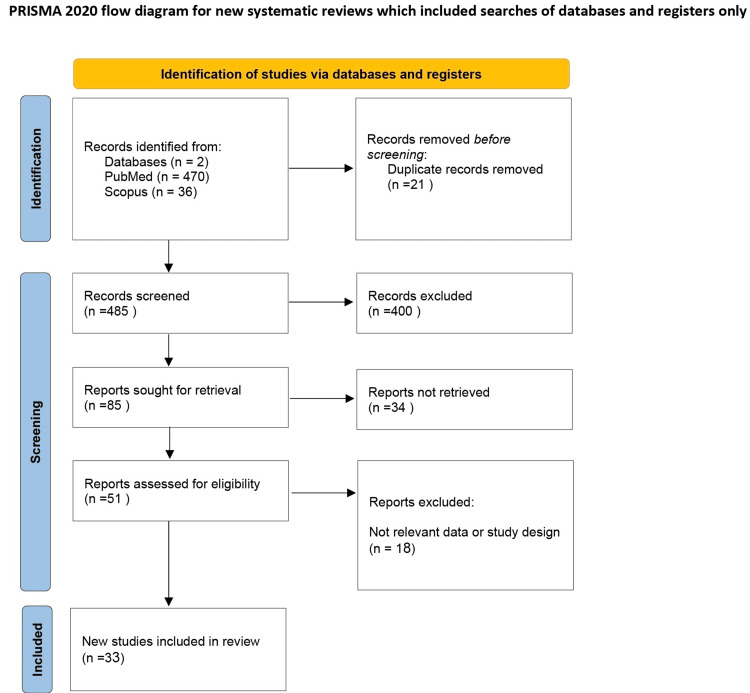
PRISMA Flow Diagram of the Included Articles. PRISMA: Preferred Reporting Items for Systematic Reviews and Meta-Analyses

A systematic review adhering to the Preferred Reporting Items for Systematic Reviews and Meta-Analyses (PRISMA) 2020 guidelines was conducted (Figure 1) [10]. We performed a comprehensive search of PubMed, Scopus, and the Web of Science from the inception of each database until December 31, 2023. The search strategy employed Boolean operators "OR" and "AND" with terms such as "carotid cavernous fistula," "CCF," "neuro-ophthalmic presentations," and "optic nerve compression". The resulting studies were uploaded to Mendeley, where duplicates were identified and removed.

Study Selection

Studies were included if they: (1) involved patients with clinically confirmed CCFs; (2) reported on clinical features, diagnostic methods, treatment approaches, and outcomes of CCFs; and (3) were written in English. Exclusions were made for studies that: (1) were reviews, book chapters, or animal and cadaver studies; (2) focused on CCFs from non-traumatic etiologies without clear clinical implications; or (3) provided insufficient clinical data on CCFs. Two reviewers (Y.A. and M.H.) independently screened the titles and abstracts of collected articles and assessed the full texts of studies meeting inclusion criteria. Any disagreements were resolved by a third reviewer (M.I.).

Data Extraction

One reviewer (M.H.) extracted the data, while two reviewers (Y. A. and M.I.) independently verified the extractions. The extracted data included authors, sample size, age, gender, clinical manifestations, venous flow dynamics, trauma history, diagnostics, treatment modalities, and outcomes. Clinical manifestations were categorized based on venous flow direction (anterior vs. posterior). Treatment responses were evaluated based on the resolution of symptoms and complications.

Data Synthesis and Analysis

Due to the heterogeneity across studies, we performed a meta-analysis in order to systematically synthesize multiple results. The main outcomes of interest were clinical presentations, diagnostic methods, treatment approaches, and patient's outcome. Table 1 details a synthesis of the key findings from all studies to offer readers an in-depth understanding of available knowledge. Our approach enables incisive knowledge of the complex and wide-ranging data characteristic of neuro-ophthalmic manifestations with CCFs through appropriate representation and analysis.

**Table 1:** Clinical Characteristics, Diagnostic Modalities, Treatment Approaches, and Outcomes of Patients with Carotid Cavernous Fistulas (CCFs) from Selected Studies.

Study Reference (Author, Year)	Sample size	Age (mean, std)	Sex (N,%)	Study Design	Location of Study	Clinical Manifestations	Venous Flow Dynamics (Anterior vs. Posterior Flow)	Trauma History (Yes/No)	Diagnostic Modality	Treatment Approach	Outcomes (e.g., Symptom resolution, Complications)	Key Findings
Kalina & Kelly, 1978 [11]	2	23.00 ± 2.00	Male (1, 50%), Female (1, 50%)	Case Report	University of Washington	Case 1: Proptosis, Neovascularization of the optic disc, Vitreous hemorrhage, Altered cranial nerve function; Case 2: Proptosis, Vitreous hemorrhage, Neovascularization of the retina	Retrograde flow through the ophthalmic artery	Yes (2)	Arteriography	Carotid artery ligations (2)	Persistent fistula (2), Neovascularization (2), Vision loss (1)	Neovascularization of the optic disc and retina post-treatment (2), highlighting the complications associated with carotid-cavernous fistula treatment
Leonard et al. 1984 [12]	15	54.14 ± 19.66	Male ( 35.8%), Female (64.2%)	Case Series	National Hospital for Nervous Diseases, Queen Square, London	Chemosis (100%), Proptosis (100%), Ophthalmoplegia (95%), Subjective bruit (70%), Reduced visual acuity (50%), Posterior segment changes (50%), Raised intraocular pressure (21%), Anterior segment ischemia (20%)	Direct CCF: Anterior(7 patient) ; Dural CCF: Posterior(8 patient)	Yes (7), No (8)	Angiography, Orbital CT	Balloon embolization (6), Spontaneous resolution (9)	Delayed recovery of the ophthalmoplegia following closure of the fistula(4), rapid recovery of ocular movements after closure of the fistula (4), biphasic recovery of ocular movements after closure of the fistula (2), no improvement in the ophthalmoplegia after closure of the fistula, although several of the other symptoms and signs regressed (4). One patient had no evidence of ophthalmoplegia associated with her direct fistula. Her symptoms of a bruit and a dull ache in the eye (1). accompanied by signs of chemosis, redness, and proptosis all resolved rapidly following embolisation of the fistula.	Different recovery rates for different types of ophthalmoplegia (10), correlation of ophthalmoplegia with muscle swelling on CT (4), no improvement in ophthalmoplegia (1)
Pérez et al, 1991 [13]	2	66.5 ± 3.5	Female (2, 100%)	Case Report	Hospital Universitario '12 de Octubre', Madrid, Spain	Case 1: Right-sided ptosis, dilated nonreactive right pupil, impaired right-eye adduction, headache, diplopia; Case 2: Left-sided ptosis, impaired left-eye adduction, headache, diplopia	Posteriorly draining dural fistula	No (2)	Carotid angiography, CT scan	Conservative management (2)	Resolution of symptoms (2)	Dural carotid-cavernous sinus fistula should be included in the differential diagnosis of painful ophthalmoplegia without other ocular signs (2)
Brosnahan et al. 1992 [14]	11	39 ± 14.6	Female (6, 54.5%), Male (5, 45.5%)	Retrospective Case Series	Institute of Neurological Sciences, Glasgow	Proptosis (11), Chemosis (10), Bruit (11), Pulsation of the globe (5), Lid engorgement (9), Venous stasis retinopathy (4), Disc oedema (2), Visual loss (2), Elevated intraocular pressure (3)	High flow (9), Moderate flow (2)	Yes (10), No (1)	Angiography	Transfemoral arterial balloon embolisation (11)	Proptosis resolution (8), Chemosis resolution (9), Bruit resolution (11), Pulsation resolution (5), Visual acuity improvement (1), Persistent visual loss (1), Intraocular pressure normalization (3), Cranial nerve palsy improvement (7)	Endoarterial balloon embolisation effectively treats traumatic carotid cavernous fistulas (11), with successful occlusion and patency of the internal carotid artery in most patients (9)
Procope et al. 1994 [15]	1	22 (Single patient case)	Male (1, 100%)	Case Report	Howard University Hospital, Washington, DC	Swelling, proptosis, decreased vision, periorbital edema, chemosis, afferent pupillary defect, reduced ocular motility, elevated intraocular pressure (26 mm Hg)	Moderate flow	Yes (1) - Previous head trauma six years prior.	CT scan, selective external carotid angiogram	Selective embolization	Rapid resolution of periorbital edema and proptosis, visual acuity stabilized at 20/200 in the right eye	Carotid cavernous sinus fistulas can present with severe symptoms without recent head trauma (1)
Acierno et al. 1995 [16]	2	63.5 ± 2.5	Female (2, 100%)	Case Report	W. K. Kellogg Eye Center, University of Michigan, Ann Arbor	Case 1: Left abduction deficit, periocular pain, horizontal diplopia; Case 2: Persistent headache, horizontal diplopia, right upper eyelid ptosis, reduced ocular motility (right eye)	Posterior drainage into inferior petrosal sinus	No (2)	Cerebral angiography, CT, MR imaging	Embolization	Resolution of pain (2), Improvement in diplopia (2)	Posterior-draining dural carotid cavernous fistulas can cause painful oculomotor palsies without congestive orbito-ocular features, requiring angiography for diagnosis (2)
Loré et al. 2003 [17]	1	67 (Single patient case)	Female (1, 100%)	Case Report	University of Siena, Azienda Ospedaliera Universitaria Senese, Siena, Italy	Moderate bilateral periorbital edema, scleral injection, conjunctival chemosis, mild proptosis, eye discomfort, excess tearing, diplopia	Dural carotid cavernous fistula draining into the ipsilateral superior ophthalmic vein	No (1) - Associated with Graves' disease	CT scan, cerebral angiography	Endovascular treatment by transvenous embolization	Immediate clinical and subjective improvement, no complications	The coexistence of Graves' ophthalmopathy and carotid cavernous fistula can lead to misdiagnosis; differential diagnosis requires reconsideration with sensitive techniques (1)
de Keizer, 2003 [18]	101	Not specified	Not specified	Clinical Research	University Hospital Antwerp, Belgium; University Medical Center Leiden, The Netherlands	Direct CCFs: Exophthalmos (96%), Specific epibulbar loops (97%), Motility disturbances (66%), Glaucoma (66%), Murmur (40%), Pulsations (15%), Ocular or orbital pain (16%), Eyelid swelling (13%), Decreased visual acuity (38%); Dural CCFs: Proptosis (93%), Specific epibulbar loops (86%), Motility disturbances (66.5%), Glaucoma (40.5%), Murmur (81%), Pulsations (52%), Headache (14%), Swelling of eyelid (12%), Decreased visual acuity (52%), Retinal hemorrhages (26%), Superior orbital fissure syndrome (10%)	Direct CCFs: High flow; Dural CCFs: Low flow	1. Direct CCFs (42): Traumatic (30), Spontaneous (12) 2. Dural CCFs (31): Traumatic (3), Spontaneous (28), 3. 10 were orbital CCFs. 4. In 18 other cases, usually dural or orbital shunts, angiography was not performed. Totally: No trauma history (68, 67%) Trauma (33, 32%)	Direct CCFs: Ultrasonography, Doppler hematotachography, Color Doppler, MRI, Angiography; Dural CCFs: Same as Direct CCFs	Direct CCFs (42): Conservative treatment (12, 7 with success; 58%), Balloon embolization (18, 17 with success; 94.5%), The other cases were treated by direct or indirect surgery, (Not specified). Dural CCFs (49): Conservative treatment (39, 32 recovered or were much improved: 82%), Embolization (7 All cured), two cases, one conservatively treated, one was embolized at another location, both with success. one case not included in the follow-up. 10 orbital arteriovenous shunts showing signs of dural fistulas, the features disappeared in 8 cases.	Direct CCFs: Conservative treatment (7 successful out of 12, 5 not successful), Balloon embolization (17 successful out of 18, 1 not successful); Dural CCFs: Conservative treatment (32 recovered or much improved out of 39, 7 not recovered or improved), Embolization (7 cured or much improved out of 7, 0 not cured or improved)	Direct CCFs: High success rate with balloon embolization, importance of follow-up with Doppler methods; Dural CCFs: Conservative treatment is often successful, importance of differentiating between progressive and diminishing clinical conditions
Ishijima et al. 2003 [19]	13 patients (14 eyes)	58 ± 14.8 (ranging from 33 to 85 years)	Male (5 eyes, 35.7%), Female (9 eyes, 64.3%)	Retrospective Multicenter Study	University of Yamanashi Faculty of Medicine, Yamanashi, Japan; Ichikawa-daimon Municipal Hospital, Yamanashi, Japan; Yamanashi Red Cross Hospital, Yamanashi, Japan	Conjunctival hyperemia (13 eyes, 92.9%), Elevated IOP (9 eyes, 64.3%), Exophthalmos (7 eyes, 50%), Retinal hemorrhage (7 eyes, 50%), Retinal vasodilation (6 eyes, 42.9%), Bruit (4 eyes, 28.6%), Hyperemia of Schlemm’s canal (3 eyes, 21.4%), External ophthalmoplegia (3 eyes, 21.4%)	Not specified	Idiopathic (12 cases), Traumatic (1 case)	CT scan, cerebral angiography	Conservative treatment (number not specified), Antiglaucoma medication (number not specified), Surgical procedures: Trabeculectomy (1), Cyclocryotherapy (1), Cyclophotocoagulation (1)	IOP control was favorable in 6 of 9 eyes with elevated IOP; 5 of these 9 eyes showed a closed CCF without requiring antiglaucoma treatment, except for 1 eye for which trabeculectomy was performed. IOP control was unfavorable in the remaining 3 eyes; 1 eye with an open CCF and 2 eyes where the CCF closure was not confirmed showed poor IOP control despite surgical and medical treatments.	Secondary glaucoma is a frequently observed ocular manifestation of CCF, and closure of the fistula is essential for favorable IOP control. The study emphasizes the importance of differential diagnosis and appropriate management of glaucoma secondary to CCF, highlighting the challenges in achieving effective IOP control.
Murata et al. 2003 [20]	1	41 (Single patient case)	Female (1, 100%)	Case Report	Department of Neurosurgery, Yokohama City University School of Medicine, Yokohama, Kanagawa, Japan	Tinnitus in the left ear, Headache, Diplopia, Pain in the left cheek, Right hemiparesis, Dysarthria, Ocular conjugate deviation to the right, Somnolence	High-flow direct carotid-cavernous fistula causing steal of blood flow from the internal carotid artery into the cavernous sinus, Occlusion of the superior petrosal sinus causing engorgement of veins in the brainstem	No (1)	Angiography, MR imaging	Embolization with interlocking detachable coils	Immediate improvement in consciousness disturbance and ophthalmoparesis, Gradual resolution of right hemiparesis and dysarthria, MR imaging showed small pontine hemorrhage and perifocal edema	Brainstem congestion caused by direct CCF is very rare but can be life-threatening. Good outcome can be expected if the CCF is completely occluded before congestive hemorrhage occurs.
Jensen et al. 2004 [21]	3	Mean 57, SD= ± 19.25	Male (2, 66.7%) Female (1, 33.3%)	Case Series	Kellogg Eye Center, University of Michigan Medical Center, Ann Arbor, Michigan, USA	Case 1: Painful red eye, diplopia, tongue numbness, jaw pain, facial droop, proptosis, lid swelling, conjunctival chemosis, reduced ocular ductions, increased IOP, facial weakness, trismus, hypesthesia of tongue and lower lip; Case 2: Diplopia, red eye, proptosis, injected and arterialized conjunctival vessels, partial abduction deficit, facial palsy, exposure keratopathy, corneal epithelial defect; Case 3: Pain behind the eye, diplopia, facial weakness, reduced ocular motility, ptosis, mydriasis, sluggish pupillary reaction	Case 1: Dural CCF with extensive filling through the meningohypophyseal trunk, occlusion of the inferior petrosal sinus; Case 2: Dural CCF fed by branches of the external carotid artery, drainage into a dilated inferior petrosal sinus; Case 3: Direct CCF with drainage into bilateral ophthalmic and inferior petrosal sinuses, stenosis of the inferior petrosal sinus	No (3)	CT, MRI, cerebral arteriography	Case 1: Endovascular closure with coils and transarterial embolization; Case 2: Transvenous embolization attempt failed, transarterial embolization with polyvinyl alcohol particles; Case 3: Transvenous catheterization and deposition of platinum coils	Case 1: Disappearance of trigeminal pain within 2 days, resolution of congestive orbitopathy within 1 week, facial weakness and trismus resolved within 2 weeks, ocular misalignment corrected within 6 weeks; Case 2: Visual acuity improvement, persistent facial palsy, and corkscrew conjunctival vessels, declined further intervention; Case 3: Immediate resolution of periocular pain, third nerve palsy resolved within 6 days, facial palsy resolved within 10 days	Facial and trigeminal neuropathies are rare manifestations of CCFs, often associated with drainage into the inferior petrosal sinus. Successful endovascular repair can lead to rapid improvement of clinical symptoms.
Peng and Liu, 2004 [22]	1	42 (Single patient case)	Female (1, 100%)	Case Report	Department of Ophthalmology, Taipei Veterans General Hospital, Taiwan	Pain, protrusion, and movement limitation of the right eye High intraocular pressure (30 mmHg) in the right eye Proptosis Abduction limitation Dilated conjunctival and episcleral vessels in the right eye Hyperemic disc	Retrograde pulsatile flow in the right superior ophthalmic vein observed with color Doppler ultrasonography	No (1)	Color Doppler ultrasonography, Carotid angiography	Gamma knife radiosurgery Peripheral retinal cryotherapy Diode laser transscleral cyclophotocoagulation	Immediate normalization of intraocular pressure (14 mmHg) and resolution of proptosis and vascular congestion Development of central retinal vein occlusion (CRVO) 5 weeks after radiosurgery, leading to neovascular glaucoma (NVG) Treated with panretinal cryotherapy and diode laser transscleral cyclophotocoagulation Eight months after gamma knife radiosurgery, color Doppler ultrasonography showed orthograde nonpulsatile flow in the right superior ophthalmic vein At the 2-year follow-up, vision was 20/600, and intraocular pressure was 12 mmHg without medication	This case highlights the potential for central retinal vein occlusion and neovascular glaucoma as complications following gamma knife radiosurgery for dural carotid-cavernous sinus fistula. Color Doppler ultrasonography can help monitor hemodynamic changes and guide treatment.
Hashimoto et al. 2005 [23]	1	79 (Single patient case)	Female (1, 100%)	Case Report	Department of Ophthalmology, Sapporo Medical University, Sapporo, Japan; Neuro-Ophthalmology Unit, Department of Ophthalmology, University of California, San Francisco, California, USA	Sudden unilateral visual loss after an ocular motor disturbance and pulsatile tinnitus, Vertical diplopia, Persistent headache, Intermittent pulsatile tinnitus in the right ear, Right facial numbness, Sudden blindness in the right eye (OD), Right oculosympathetic paresis, third, sensory fifth, and sixth cranial nerve pareses, Inwardly deviated right eye (OD), Relative afferent pupillary defect (OD), Mild right upper lid ptosis, Hypesthesia in all three divisions of the right trigeminal nerve	Posterior-draining dural carotid cavernous sinus fistula fed by the right meningohypophyseal trunk and right middle meningeal artery, Ophthalmic-middle meningeal arterial anastomosis	No (1)	Selective angiography of the right internal and external carotid arteries, Serial dynamic MRI	Transvenous and transarterial catheters depositing venous coils and arterial polyvinyl alcohol particles into the fistula	One month after embolization: Right oculosympathetic paresis resolved, Partial resolution of third and sixth cranial nerve deficits, Persistent blindness in the right eye (OD)	This case highlights that posterior ischemic optic neuropathy can occur in posterior-draining dural CCF due to arterial steal phenomenon. Early diagnosis and embolization can resolve cranial nerve deficits, though visual recovery may not always be achieved.
Ikeda et al. 2005 [24]	1	55 (Single patient case)	Female (1, 100%)	Case Report	Kagawa University School of Medicine, Kagawa, Japan	Intractable right orbitofrontal headache, Diplopia due to right abducens nerve palsy, No orbito-ocular signs observed throughout the clinical course	Dural carotid-cavernous sinus fistula with three directional drainage routes in the arterial phase, Prominent drainage into the superior ophthalmic vein (SOV), Outflow with a high flow rate into the angular facial vein, preventing prolonged enhancement of the SOV in the venous phase	No (1)	Brain MRI, MR angiography, Angiography	Endovascular embolization with coils via the facial vein and SOV	Gradual resolution of abducens nerve palsy over 2 months	The absence of orbito-ocular signs in dural CCF with anterior venous drainage could be attributed to the relief of venous hypertension of the SOV due to high flow rate outflow into the angular facial vein.
van Rooij et al. 2006 [25]	11	61.6 ± 15.2 (ranging from 27 to 77 years)	Female (8, 72.7%), Male (3, 27.3%)	Original Research	St. Elisabeth Ziekenhuis, Tilburg, the Netherlands	Audible pulsatile bruit (11, 100%), Bilateral exophthalmus and ophthalmoplegia (7, 63.6%), Decreased vision (8, 72.7%), Hemiplegia and aphasia (1, 9.1%), Major cortical venous drainage associated with intracranial hemorrhage (2, 18.2%)	High-flow CCFs: 5 cases, Intermediate-flow CCFs: 3 cases, Low-flow CCFs: 3 cases, Venous drainage to superior ophthalmic veins, minor cortical venous drainage	No (11)	Angiography, MRI	Coil occlusion of the aneurysm: 5 cases, Balloon occlusion of the aneurysm: 1 case, Coil occlusion of both aneurysm and internal carotid artery: 2 cases, Spontaneous closure of low-flow CCFs: 2 cases (subsequently treated with coil and balloon occlusion)	Visual acuity returned to normal in all but one patient, Ophthalmoplegia cured in 6 of 8 patients, Remaining abducens nerve palsy corrected surgically in 2 patients, One patient died before treatment	The incidence of CCF caused by a ruptured cavernous sinus aneurysm was 1.5%. Clinical symptoms correlate with venous drainage. Drainage to cortical veins may lead to intracranial hemorrhage. Endovascular treatment with coils is effective in occluding the fistula.
Wu et al. 2006 [26]	11	65.4 ± 12.1 (ranging from 39 to 80 years)	Female (9, 81.8%), Male (2, 18.2%)	Retrospective Study	Department of Neurology, Chang Gung Memorial Hospital, Taipei, Taiwan; Department of Radiology, E-Da Hospital/I-Shou University, Kaohsiung County, Taiwan	Oculomotor nerve palsy: Eight cases (72.7%), Abducens nerve palsy: Two cases (18.2%), Trochlear nerve palsy: One case (9.1%), Associated symptoms: Headache or ocular pain (7 cases, 63.6%), No congestive ocular features	All cases involved posterior-draining DCCFs: Draining veins: Superior petrosal sinus, inferior petrosal sinus, basilar venous plexus, pterygoid plexus	No (11)	Brain CT/CT angiography (CTA): four patients (all unremarkable), MRI/MR angiography (MRA): nine patients (6 showed compatible findings of DCCF), Digital subtraction angiography (DSA): Confirmed diagnosis in all cases	Conservative treatment with carotid compression: five cases, Transarterial embolization (TAE): three cases, Transvenous embolization (TVE): two cases, Radiotherapy: one case	Complete recovery in six cases (54.5%) within 12 months, Mild residual ophthalmoplegia in 4 cases, Recurrence observed in three cases (successfully obliterated after subsequent TAE, but one case resulted in residual left eye blindness)	DCCFs with isolated ocular motor nerve palsy are not uncommon. MRI/MRA is useful for initial evaluation, but DSA is necessary for accurate diagnosis and treatment planning. Posterior drainage is associated with ocular motor nerve palsy without congestive ocular features.
Das et al. 2006 [27]	1	58 (Single patient case)	Male (1, 100%)	Case Report	Northwestern University Feinberg School of Medicine, Chicago, IL, USA; University of Chicago Pritzker School of Medicine, Chicago, IL, USA	Right eye visual loss progressing to complete blindness over 5 weeks post-carotid artery angioplasty and stenting, Right proptosis, retro-orbital pain, right facial numbness, pulsatile proptosis, diminished visual acuity, occasional diplopia, Complete right eye visual loss, including loss of light perception, paralysis of extraocular muscles	High-flow direct CCF confirmed by angiography, with dissection of the proximal right ICA extending to the cavernous portion, Engorgement of superior and inferior ophthalmic veins	No (1)	Brain MRI, CT angiography, Digital subtraction angiography (DSA)	Transarterial coil embolization of the cervical and cavernous carotid segments	Significant reduction in proptosis and injection of the affected eye on postprocedure day 1, Regained some extraocular muscle function and light perception in right eye on postprocedure day 5, Visual acuity improved to 20/100 by discharge on day 10, and further improved to 20/50 by postprocedure day 60	This case demonstrates that even with early morphological changes associated with prolonged retinal ischemia, aggressive endovascular intervention can result in recovery of vision in patients with CCF.
Théaudin et al. 2008 [28]	1	75 (Single patient case)	Female (1, 100%)	Case Report	AP-HP, Hôpital Lariboisière, Paris, France	Severe frontal headache, Transient diplopia, Recurrent diplopia, Bilateral conjunctival injection, Bilateral episcleral and conjunctival hyperemia, Vertical paresis of the left eye, Complete ophthalmoplegia and ptosis of the right eye, Partial motor seizures, Right facial palsy, Aphasia	DCCF draining into leptomeningeal veins, Outflow veins: left inferior petrosal sinus draining into the internal jugular vein, left superficial sylvian vein draining into cortical veins in the left temporal and parietal lobes, Occlusion of right inferior petrosal sinus	No (1)	Brain CT, MR scans, T2-weighted MRI, Conventional angiogram, Gradient echo MRI, Gadolinium-enhanced angio-MRI	Percutaneous transvenous embolization via the left internal jugular vein to the left cavernous sinus, then to the right cavernous sinus, Occlusion of the right cavernous sinus with coils	Hemiplegia and worsened aphasia post-embolization due to cortical brain hemorrhage, Resolution of ophthalmologic symptoms within a few days, Hemiplegia resolved within 1 month, Moderate aphasia persisted at 1 year follow-up	This case highlights the potential for cortical brain hemorrhage after technically successful embolization of DCCF draining into leptomeningeal veins. Monitoring and management of venous drainage changes are crucial to prevent complications.
Grumann et al. 2012 [29]	47	55.78 ± 20.73 (ranging from 13 to 89 years)	Female (28, 57.8%), Male (19, 42.2%)	Retrospective Study	University Hospital Center Dupuytren, Limoges, France; Hôpital Lariboisière, Paris, France	Blurred vision: 17 patients (36.2%), Proptosis: 37 patients (78.7%), Headaches: 14 patients (29.8%), Eye redness: 32 patients (68.1%), Ophthalmoparesis: 29 patients (61.7%), Chemosis: 20 patients (42.6%), Bruit: 17 patients (36.2%), Elevated IOP: 15 patients (31.9%), Oculomotor nerve (III) involvement: 24 patients (19.1% isolated, 31.9% multiple), Abducens nerve (VI) involvement: 5 patients (10.64%)	Direct CCF (21 patients): Lower average age, associated with encephalic trauma in 13 patients (61.9%), Indirect CCF (26 patients): Higher average age, associated with encephalic trauma in 5 patients (19.2%), Drainage sites: superior ophthalmic vein, petrosal sinuses, cortical veins	Yes (18), No (29)	Bilateral selective ICA and ECA angiographies	Endovascular treatment (venous or arterial embolization), Repeat embolizations performed as necessary	Persisting ophthalmologic signs/symptoms in 19 patients (40.4%) after endovascular treatment, Diplopia and oculomotor muscle paresis more frequent in patients with direct CCF, Significant association between initial decrease of visual acuity and persistence of ocular symptoms (odds ratio 3.33)	Elevated IOP significantly associated with indirect CCFs, Murmur significantly associated with direct CCFs, Initial decrease in visual acuity linked to worse ophthalmic prognosis
Nguyen et al. 2013 [30]	1	34 (Single patient case)	Male (1, 100%)	Case Report	Department of Reconstructive and Maxillofacial Surgery, Vietnam-Cuba Hospital, Hanoi, Vietnam; Department of Plastic and Reconstructive Surgery, Ajou University Hospital, Suwon, Korea	Tinnitus, Bruit in the right orbital area, Diplopia, Eye pain, Headache, Severe limitation of ocular movement on lateral gaze	Dilated superior ophthalmic vein, CCF with cranial nerve VI palsy	Yes (1)	Brain angio-CT	Endovascular coiling embolization	Immediate reduction in bruit and tinnitus post-procedure, Significant improvement in cranial nerve VI palsy and related symptoms (diplopia and limited ocular movement) within 4 months post-coiling	This case illustrates the importance of careful follow-up for patients with skull base fractures to rule out CCF. Delayed presentation of symptoms (up to 8 weeks post-injury) can occur and should be considered in the differential diagnosis.
Kim et al. 2013 [31]	1	32 (Single patient case)	Female (1, 100%)	Case Report	Department of Oral and Maxillofacial Surgery, Ewha Womans University, Seoul, Republic of Korea	Bilateral blowout fractures, Diplopia with impaired abduction of the left eye, No orbito-ocular signs such as exophthalmos, ptosis, or chemosis	Posteriorly draining CCF with isolated abducens nerve palsy	Yes (1)	Carotid angiography	Coil embolization	Full resolution of ophthalmoplegia post-coil embolization	This case highlights the potential for missed diagnosis of posteriorly draining CCFs, termed 'white-eyed shunt,' which lack classic congestive orbital signs. Early recognition and treatment are crucial to prevent morbidity.
Tan et al. 2014 [32]	45	Average age for: 1. Direct CCF (n=8) is 41(18–54). 2. Dural CCFs (n = 37) is 58(31–82).	Female (26, 57.8%), Male (19, 42.2%)	Retrospective Study	Singapore National Eye Centre, Singapore; Singapore Eye Research Institute, Singapore; National University of Singapore, Singapore; Interventional Radiology Department, Singapore General Hospital, Singapore	Anterior draining CCFs: Orbital congestion, Posterior draining CCFs: Pain, diplopia, cranial nerve palsies, Mild residual symptoms in 85% of treated direct CCFs despite complete angiographic closure, 52% of treated dural CCFs had complete resolution of symptoms despite only half achieving angiographic closure, Common symptoms before treatment: Visual acuity worse than 6/60: 6% (2 patients), Raised intra-ocular pressure (IOP) > 21 mmHg: 34% (11 patients), Significant proptosis: 42% (12 patients), Diplopia: 56% (18 patients)	Direct CCFs: Often associated with trauma, presented with orbital congestion, Dural CCFs: Could be asymptomatic or present with cranial nerve palsies without orbital congestion, could drain anteriorly or posteriorly	Yes(totally 9, 20%) (7 for direct CCF, 2 for dural CCF), No (36)	Conventional cerebral angiography (gold standard), CT, MRI, Doppler ultrasound	Endovascular embolization and observation, Treated cases: Direct CCFs: 7 treated, 1 observed, Dural CCFs: 29 treated, 8 observed	After treatment: Visual acuity worse than 6/60: 9% (3 patients), Raised IOP > 21 mmHg: 9% (3 patients), Significant proptosis: 11% (3 patients), Diplopia: 16% (5 patients), Poor clinical outcomes more associated with patients on anticoagulant/antithrombotic medications and those presenting with poor visual acuity (<6/60)	Presenting symptoms and signs are related to the angiographic drainage of CCFs. Angiographic outcomes after embolization treatment may not always correlate with clinical outcomes. Elevated IOP significantly associated with indirect CCFs, while murmurs were more associated with direct CCFs. Initial decrease in visual acuity linked to worse ophthalmic prognosis.
Erickson and Johnson, 2014 [33]	1	32 (Single patient case)	Male (1, 100%)	Case Report	Department of Ophthalmology, Bascom Palmer Eye Institute, Miami, Florida, USA	Proptosis, Ocular motility deficits, Decreased vision (right eye), Orbital bruit, Slight right exotropia, Moderate motility deficits in all cardinal gaze directions, Dilated conjunctival blood vessels, 3 mm of proptosis in the right eye	Profound dilatation of the right superior ophthalmic vein	Yes (1)	Digital subtraction angiography	Covered stent and embolic agent used to abolish arteriovenous communication	Rapid return to premorbid baseline, Improved visual acuity to 20/20, Normalized intraocular pressures, Resolution of motility deficits and double vision, Resolution of proptosis	This case highlights the importance of recognizing CCF in patients with craniofacial trauma presenting with exophthalmos, arterialized conjunctival vessels, and orbital bruit. Early intervention with endovascular techniques can lead to rapid and complete recovery.
Leishangthem and Satti, 2017 [34]	1	71 (Single patient case)	Female (1, 100%)	Case Report	Albert Einstein Medical Center, Philadelphia, Pennsylvania, USA; Christiana Care Health Center, Newark, Delaware, USA	Progressive left-sided monocular diplopia and ptosis, Initially diagnosed as monocular myasthenia gravis, Left-sided proptosis, Ocular bruit, Partial third and fourth nerve palsies, Chemosis of left eye inferiorly, Corkscrewing of conjunctival blood vessels	Indirect high-flow left CCF, type D (supply from both ICA/ECA meningeal branches)	No (1)	MRI, MRA, Conventional cerebral angiogram, Digital subtraction angiography	Endovascular treatment with Onyx liquid and platinum coil embolization	Complete resolution of ophthalmologic symptoms, Residual left arm and hand hemiparesis and dysmetria secondary to a brachial plexus injury	This case illustrates the importance of considering indirect CCF as a differential diagnosis in patients with progressive ocular symptoms. Early recognition and intervention can prevent unnecessary treatments and lead to better outcomes.
Zhu et al. 2018 [35]	1	22 (Single patient case)	Male (1, 100%)	Case Report	Department of Ophthalmology, The First Affiliated Hospital, Jinan University, Guangzhou, China	Left eyelid swelling, eye redness, visual decrease, occasional headache, Initially thought to have glaucoma, Left eye symptoms: Blurred vision, swelling, hyperaemia, mild ptosis, exophthalmos, chemosis, corkscrew hyperaemia centred on the cornea, Right eye symptoms: Slight hyperaemia	Direct unilateral CCF with symptoms in the contralateral eye MRI revealed broadening of the left superior ophthalmic vein, slight thickening of the left lateral rectus muscle, expansion of the left cavernous sinus	Yes (1)	Magnetic resonance imaging (MRI), digital subtraction angiography (DSA)	Detachable balloon catheter embolization surgery	All symptoms resolved post-surgery, including redness and swelling of the left eye, blurred vision, and double vision Visual acuity improved to 5/5 in the left eye, intraocular pressure normalized to 18 mmHg	This case is the first reported of a post-traumatic unilateral CCF with contralateral symptoms in direct CCF. It highlights the importance of maintaining a high suspicion of CCF and confirming the diagnosis by DSA to prevent serious consequences.
McManus et al. 2018 [36]	1	49 (Single patient case)	Male (1, 100%)	Case Report	Emergency Department, Mercy Health Partners, Muskegon, Michigan, USA	Left-sided retro-orbital pain Blurred vision Binocular diplopia with rightward gaze Photophobia Unilateral left-sided proptosis Conjunctival chemosis Anisocoria with a pupil 2 mm larger on the left Reduced extraocular movements in all directions Decreased visual acuity to finger counting in the affected eye Audible bruit over the affected eye No vitreous hemorrhage or papilledema on fundoscopic examination Slit-lamp examination showed limbal flare but no hyphema Intraocular pressure: 16 mm Hg bilaterally	Left-sided proptosis with marked prominence of the left superior ophthalmic vein and additional extraconal veins medially within the left orbit	Yes (1) - fell 15 feet out of a tree stand 2 months prior	Maxillofacial CT Cerebral angiography	Urgent cerebral angiography and definitive endovascular neurosurgical intervention (coil embolization of the fistulous tracts)	Complete resolution of visual symptoms within 3 weeks post-treatment	The case highlights the importance of recognizing CCF in patients with craniofacial trauma presenting with exophthalmos, arterialized conjunctival vessels, and orbital bruit. Early intervention with endovascular techniques can lead to rapid and complete recovery.
Lin and Hu, 2019 [37]	1	32 (Single patient case)	Male (1, 100%)	Case Report	Department of Ophthalmology, Taipei City Hospital, Renai Branch, Taipei, Taiwan	Progressive double vision for 4 months Right-sided headache and periocular pain Right-side partial ptosis Mid-dilated right pupil with poor reaction to light Limited right-side extraocular movement with impaired adduction and infraduction No chemosis, proptosis, conjunctival injection, swollen eyelids, or ocular bruits Normal best-corrected visual acuity and intraocular pressure of both eyes Normal other cranial nerve functions	Right-sided CCF primarily supplied by the dural branch of the right middle meningeal artery with venous drainage into the right inferior petrosal sinus	Yes (1) - minor head trauma from a road accident 1 year prior	Time-of-flight magnetic resonance angiography (TOF-MRA) Digital subtraction angiography (DSA)	Transarterial coil embolization	Significant improvement in right-side headache and partial resolution of ptosis and limited extraocular muscle movement at 2 months post-treatment	CCF may not always present with ocular congestion. White-eye and painful third nerve palsy with pupillary involvement can be caused by posterior drainage CCF. Early MRI/MRA should be performed to exclude aneurysms and other causes.
Azzam et al. 2021 [38]	1	29 (Single patient case)	Male (1, 100%)	Case Report	Department of Ophthalmology, Division of Oculofacial Plastic & Orbital Surgery, Gavin Herbert Eye Institute, University of California, Irvine, Irvine, USA	Right conjunctival injection Progressive proptosis Diplopia Right-sided proptosis, periorbital edema, and conjunctival injection Generalized ophthalmoplegia of the right eye most noticeable on abduction Tortuous episcleral vessels and blood in Schlemm’s canal Dilated, tortuous retinal vessels	Cavernous sinus enhancement on CT Low-flow indirect carotid-cavernous fistula (CCF) with flow reversal into the right superior ophthalmic vein	Yes (1) - minor motor vehicle crash 4 years earlier	Digital subtraction angiography (DSA) Computed tomography (CT)	Endovascular embolization with the liquid embolic system, Onyx® (EV3, Irvine, CA, USA)	Improvement in conjunctival injection and abduction one day post-operation Dramatic improvement in proptosis at three weeks post-operation Resolution of motility deficit and conjunctival injection at seven weeks post-operation No central nervous system, orbit, or ocular issues at six months follow-up	This case highlights the importance of considering CCF in patients with symptoms mimicking thyroid eye disease (TED), especially with a smoking history. Detailed ophthalmologic examination and neuroimaging are crucial for accurate diagnosis and timely intervention.
Cavasin et al. 2021 [39]	1	68 (Single patient case)	Female (1, 100%)	Case Report	Department of Neuroradiology, Ospedale dell’Angelo, Mestre, Italy	Left retro-orbital pain Lacrimation Visual disturbance Redness and edema of the conjunctiva Spontaneous retrobulbar pain and pain on up or down gaze Subjective intermittent diplopia due to eye motility impairment No signs of optic nerve involvement such as visual acuity loss No significant hypertrophic superior or inferior ophthalmic vein	Left exophthalmos Global left extrinsic ocular muscles increase in size without significant hypertrophic superior or inferior ophthalmic vein	No (1)	Orbital MRI Intracranial CT angiography Digital subtraction angiography (DSA)	Initial transvenous endovascular embolization attempt failed Subsequent steroid treatment regimen for moderate-to-severe ophthalmopathy (intravenous methylprednisolone and oral prednisone)	Venous pouch of the CS-DAVF progressively decreased in size and was completely occluded after the cycle of corticosteroid therapy Proptosis regressed, diplopia and retroorbital pain disappeared Slight conjunctival hyperemia remained Patient free of symptoms at three-year follow-up	The case suggests that some cases of CS-DAVF may be secondary to ocular muscle hypertrophy, and treating the ocular disease with medical therapy may resolve the vascular problem as well. This is a rare instance of complete resolution of a dural fistula with sole medical therapy.
Agrawal et al. 2022 [40]	1	60 (Single patient case)	Female (1, 100%)	Case Report	Department of Ophthalmology, Command Hospital, Pune, India; Department of Ophthalmology, Armed Forces Medical College, Pune, India	Left-sided headache Pain, protrusion, and redness of the left eye Vision of 20/80 Proptosis Chemosis Severe ophthalmoplegia Dilated cork-screw tortuous episcleral vessels in the left eye No cells/flare in anterior chamber, normal posterior segment, normal right eye examination	Low-flow left indirect CCF Increase in bulk of all extraocular muscles with tendon sparing on MRI	No (1)	Digital subtraction angiography (DSA) Magnetic resonance imaging (MRI) Optical coherence tomography (OCT)	Transvenous embolization of the CCF	Complete obliteration of the CCF post-embolization Dramatic resolution of ocular symptoms in three days Vision improved to 20/20 one week post-embolization Ocular symptom-free at three months follow-up	This case highlights the importance of considering CCF in patients with symptoms mimicking thyroid-associated orbitopathy (TAO). Detailed ophthalmologic examination and neuroimaging are crucial for accurate diagnosis and timely intervention.
Pellegrini et al. 2022 [41]	1	92 (Single patient case)	Female (1, 100%)	Case Report	Department of Ophthalmology, Santo Spirito Hospital, Pescara, Italy; Azienda Sanitaria Locale (ASL) Pescara, Pescara, Italy; Villa Anna Hospital, San Benedetto, Italy; Asur Marche Area Vasta 3, Macerata, Italy; Houston Methodist Hospital, Houston, USA	Bilateral eye redness Lid fullness Conjunctival chemosis Ophthalmoplegia Ptosis Bilateral proptosis Severe conjunctival chemosis and congestion Almost complete bilateral ophthalmoplegia Complete right superior eyelid ptosis Best-corrected visual acuity: 20/200 OU Intraocular pressure: 21 mmHg OU Mild engorgement of retinal veins with no optic disc swelling	Bilateral dilation of the superior ophthalmic veins Direct high-flow carotid-cavernous fistula with secondary extraocular muscle enlargement	No (1)	Computed tomography (CT) Computed tomography angiography (CTA)	The patient chose to be treated in a nearby hospital and was lost on follow-up	No follow-up data available	Clinicians should be aware that direct high-flow CCFs, although usually occurring after trauma and unilaterally, can present spontaneously without trauma and bilaterally. Spontaneous direct CCFs should be considered in patients presenting with acute changes in vision, headache, and proptosis regardless of the history of trauma. The distinctive radiographic sign on CT or MRI is dilation of the SOV. Standard catheter angiography is typically necessary both for diagnosis and treatment with endovascular embolization.
Krothapalli et al. 2023 [42]	1	56 (Single patient case)	Male (1, 100%)	Case Report	Department of Neurology, University of Connecticut, Farmington, CT, USA; Departments of Interventional Neuroradiology and Neurosurgery, Hartford Hospital, Hartford, CT, USA	Progressive right eye proptosis Congestion Decreased visual acuity (20/40) Limited duction Exophthalmos Pulsatile tinnitus Elevated intraocular pressure No optic disc swelling Significant inflammation involving the right orbit and atypical enhancement of the basal frontal lobe adjacent to the orbit	Enlarged superior ophthalmic vein Enlarged frontal vein Indirect right carotid cavernous fistula (CCF) Right sigmoid sinus thrombosis with stenosis of the right internal jugular vein	No (1)	Magnetic resonance imaging (MRI) Magnetic resonance angiography (MRA) with time-of-flight Conventional cerebral angiogram	Endovascular treatment with transvenous approach through the right inferior petrosal sinus using a coil and Onyx liquid embolic agent	Remarkable improvement in symptoms post-treatment No evidence of right eye congestion, exophthalmos, elevated intraocular pressure, or vision changes post-treatment	Clinicians should be aware that CCFs can present without clear predisposing factors and can be secondary to conditions such as sigmoid sinus thrombosis. Early recognition and timely intervention are crucial for resolving orbital hypertension-related symptoms. Conventional cerebral angiography is necessary for diagnosis and treatment guidance.
Yan et al. 2024 [43]	120	Totally: Mean: 44.2, SD= ±10.8 Direct CCF: Mean=35.65 SD=±5.4 Indirect CCF: Mean=54.6 SD=± 5.5 Mixed CCF: Mean=42.4 SD=± 12.5	Totally Male (63, 52.5%), Female (57, 47.5%) Direct CCF: 86 Male: 53 (61%) Female:33(38%) Indirect CCF: 23 Male:3(13%) Female:20(87%) Mixed CCF: 11 Male:7(63%) Female:4( 36%)	Retrospective Cohort Study	Department of Ophthalmology and Visual Sciences, College of Medicine and Philippine General Hospital, University of the Philippines Manila	Direct CCF: Proptosis: 49 Eye redness: 21 Diplopia: 7 Blurring of vision: 3 Eyelid swelling: 3 Eye pain: 3 Upper lid mass: 0 Indirect CCF: Proptosis: 9 Eye redness: 8 Diplopia: 2 Blurring of vision: 1 Eyelid swelling: 2 Eye pain: 0 Upper lid mass: 1 Mixed CCF: Proptosis 7 Eye redness 1 Diplopia 1 Blurring of vision 2 Eyelid swelling 0 Eye pain 0 Upper lid mass 0	Dilated superior ophthalmic vein: 103 Proptosis: 80 Enlarged extraocular muscles: 65	Totally: Trauma (80, 66.6%) Direct CCF: 67(83.7%) Indirect CCF: 6(7.5%) Mixed CCF: 7(9%)	Computed tomography (CT) Magnetic resonance imaging (MRI) Cerebral angiography (Gold standard)	Group A: No endovascular treatment (109 patients) Group B: Endovascular treatment (11 patients)	Group A: Spontaneous improvement or worsening of clinical features over time Group B: Improvement or no worsening of features after endovascular treatment Odds Ratios: Visual Acuity: 1.95 (0.40, 9.52) Corkscrewing of conjunctival vessels: 6.25 (1.31, 29.80) Proptosis: 3.87 (1.06, 14.14) Intraocular Pressure: Not statistically significant Extraocular movement limitation: 3.35 (0.84, 13.32) Diplopia: 0.87 (0.21, 3.51) Bruit audible by patient: Not statistically significant Pulsation: 1.78 (0.21, 14.86)	Endovascular treatment is effective and safe for improving visual acuity, corkscrewing of conjunctival vessels, proptosis, extraocular movement limitation, diplopia, and audible bruit. Direct CCFs are more common in males and younger individuals with trauma as the major risk factor. Indirect CCFs are more common in females and older individuals with hypertension as the major risk factor. Clinical presentation varies between direct and indirect CCFs, with direct CCFs having a more acute presentation. Radiologic findings are crucial for diagnosis, with cerebral angiography being the gold standard.

Results

Study Selection

This comprehensive analysis includes 33 studies involving 403 individuals with CCFs (Table 1). The reviewed studies covered clinical aspects, diagnostic methods, treatment techniques, and outcomes.

Patient Demographics

The mean age of patients with direct CCFs was 42.99 years, whereas those with indirect CCFs averaged 55.88 years. Direct CCFs were more prevalent in males, with 102 out of 197 patients (51.56%) being male and 95 (48.11%) females. Conversely, indirect CCFs were more common in females, comprising 101 out of 179 patients (56.44%) compared to 78 (43.56%) males. Detailed demographics and clinical characteristics are presented in Table 2.

**Table 2:** Patient Demographics and Clinical Characteristics of Carotid Cavernous Fistulas. CCFs: Carotid cavernous fistulas

Characteristics	Direct CCFs (N=197)	Indirect CCFs (N=179)	Total (N=403)
Mean age (years)	42.99	55.88	
Gender distribution			
- Male (%)	102 (51.56%)	78 (43.56%)	180 (47.87%)
- Female (%)	95 (48.11%)	101 (56.44%)	196 (52.13%)
Clinical manifestations			
- Proptosis	49(24.87%)	9 (5.03%)	58 (14.39%)
- Eye redness	21 (10.66%)	8 (4.47%)	29 (7.20%)
- Diplopia	7 (3.55%)	2 (1.12%)	9 (2.23%)
- Blurred vision	3 (1.52%)	1 (0.56%)	4 (0.99%)
- Eyelid Swelling	3 (1.52%)	2 (1.12%)	5 (1.24%)
- Eye pain	3 (1.52%)	0 (0%)	3 (0.74%)
- Upper lid mass	0 (0%)	1 (0.56%)	1 (0.25%)
Trauma history			
- Yes (%)	67 (34.01%)	6 (3.35%)	73 (18.11%)
- No (%)	19 (9.64%)	17 (9.50%)	36 (8.93%)

Clinical Manifestations

**Figure 2:**
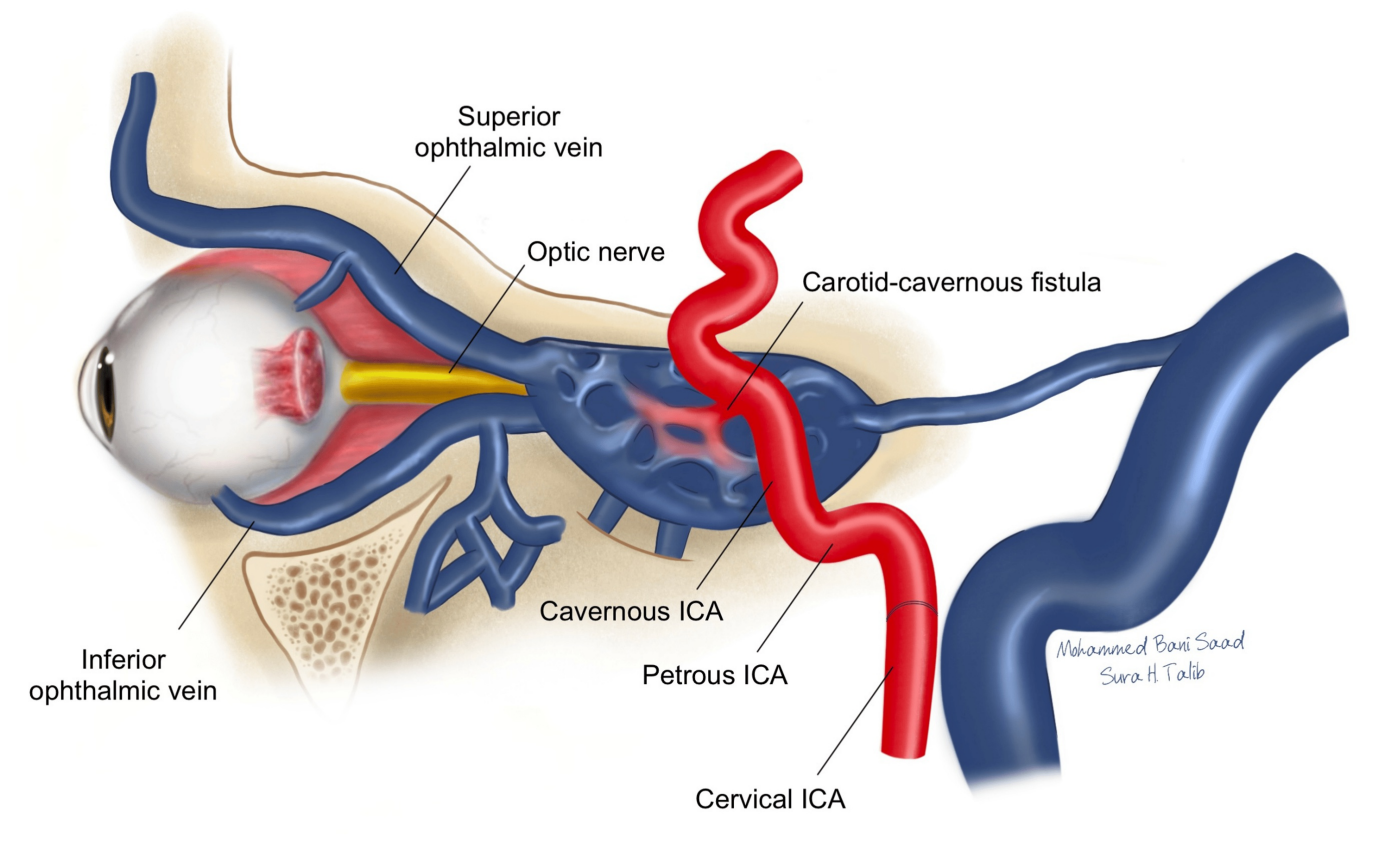
Anatomical Illustration of the Carotid Cavernous Fistula (CCF). ICA: Internal carotid artery Image Credit: Sura H. Talib

The clinical manifestations varied significantly between direct and indirect CCFs. For direct CCFs, common symptoms included proptosis in 49 cases (24.87%), eye redness in 21 patients (10.66%), double vision in seven patients (3.55%), blurred vision in three patients (1.52%), eyelid swelling in three patients (1.52%), and eye pain in three patients (1.52%) (Table 1). These symptoms are due to abnormal venous pressure and congestion in the orbital area (Figure 2). Indirect CCFs presented with proptosis in nine patients (5.03%), eye redness in eight patients (4.47%), diplopia in two patients (1.12%), blurred vision in one patient (0.56%), eyelid swelling in two patients (1.12%), and an upper lid mass in one patient (0.56%) (Table 1). Overall, combined manifestations among all patients with CCFs included proptosis in 58 cases (14.39%), eye redness in 29 patients (7.20%), diplopia in nine patients (2.23%), blurred vision in four patients (0.99%), eyelid swelling in five patients (1.24%), eye pain in three patients (0.74%), and an upper lid mass in one patient (0.25%). Patients with anterior venous flow primarily exhibited proptosis and eye redness due to the arterialization of the superior and inferior ophthalmic veins. In contrast, those with posterior venous flow often experienced cranial nerve VI (abducens) palsy, resulting in double vision, and cranial nerve V (trigeminal) involvement, causing facial pain or headache.

Venous Flow Dynamics

Venous flow dynamics were characterized by dilated superior ophthalmic veins in 103 instances (25.56%), proptosis in 80 cases (19.85%), and enlarged extraocular muscles in 65 cases (16.13%) (Table 1).

Trauma History

A significant factor for direct CCFs was trauma history, with 67 out of 197 patients (34.01%) having experienced trauma. Conversely, indirect CCFs were frequently associated with trauma history, observed in 6 out of 179 patients (3.35%) (Table 2).

Diagnostic Modalities

The primary diagnostic techniques used included CT, MRI, and cerebral angiography, which is considered the gold standard. These imaging methods were crucial for confirming the diagnosis and devising treatment plans (Table 1).

Treatment Approaches

Patients were treated by conservative, surgery and endovascular methods. Fistula closure was achieved using endovascular techniques such as coil and Onyx embolization, allowing the blood flow to return into its normal pathway. These methods are where patient results have been drastically improved in the treatment of CCFs. Management is multimodal and it depends on various factors including CCF features and patient-related health issues (Table 1).

Outcomes

Outcomes for patients varied; some experienced spontaneous improvement, while others reported a progression of symptoms. Those who underwent endovascular therapy generally showed significant clinical improvement, without deterioration of symptoms. Significant outcomes included enhanced visual acuity, reduced conjunctival vessel corkscrewing, decreased proptosis, and resolved diplopia (Table 1).

Key Findings

Direct CCFs were more common in younger males with a history of trauma, while indirect CCFs were prevalent in older females with hypertension. Endovascular treatment proved effective for managing symptoms and halting the progression of CCFs. Early diagnosis and intervention are critical for achieving better patient outcomes (Table 1).

Discussion

This review comprehensively details the wide-ranging neuro-ophthalmic symptoms associated with CCFs and underscores the necessity for timely diagnosis and intervention.

Clinical Presentations

The clinical manifestations of CCFs are pivotal for diagnosing and treating this condition. Our data analysis, encompassing both direct and indirect CCFs, reveals significant trends in symptom distribution.

Proptosis was the most frequently observed symptom, present in 53.6% of cases. This symptom should raise immediate concern for CCFs, especially in patients with sudden onset. Proptosis, common in both direct and indirect CCFs, indicates disrupted venous drainage in the orbit, resulting in increased orbital pressure [11,12,44].

Redness in the eyes was the second most common symptom, seen in 39.3% of cases. This redness, caused by congestion due to elevated venous pressure, needs to be distinguished from conjunctivitis or uveitis [32,44].

Diplopia, affecting 29% of patients, reflects cranial nerve palsy related to eye movement, highlighting the neuro-ophthalmic complications of CCFs and the risk of long-term disability if untreated. Comprehensive neuro-ophthalmic exams are essential in suspected CCF cases due to the high prevalence of diplopia.

Blurred vision, reported by 23.2% of patients, suggests potential optic nerve involvement and retinal ischemia, posing a risk of permanent vision loss if not addressed. This symptom's prevalence necessitates early imaging and intervention [13-17,45].

Eyelid swelling, occurring in 16.3% of cases, signals significant orbital congestion and must be differentiated from orbital cellulitis and other inflammations. Accurate diagnosis is crucial for proper treatment [46].

Ocular pain, though less common at 7.5%, significantly impacts patient quality of life. This pain likely results from increased intraocular pressure and ischemia. Recognizing this symptom early can enhance patient comfort and prevent further complications [28-32].

Upper lid mass, the least common symptom at 4%, remains a notable clinical feature. Despite its rarity, it can aid in diagnosing CCFs when it is present with other symptoms.

Diagnosis

DSA remains the gold standard for diagnosing CCFs, as it visualizes fistulous connections and blood flow dynamics. Non-invasive imaging methods like CT, MRI, and MRA are invaluable for initial assessment and follow-up, providing essential information on vascular involvement to guide targeted therapies.

Treatment Approaches

Endovascular treatments are now the first-line therapy for CCFs due to their low invasiveness and high success rates. Techniques such as embolization with coils, Onyx, and other agents via trans-arterial or transvenous routes effectively close the fistula and alleviate symptoms. These strategies not only address immediate clinical concerns but also protect patients from severe complications.

Limitations and future directions

While this review has strengths, it is important to acknowledge limitations, including heterogeneous studies with varying sample sizes, diagnostic criteria, and treatment protocols. The retrospective design of most studies introduces potential bias and limits causal evaluation. Future prospective studies with standardized protocols are needed to improve comparability and provide stronger evidence. Advancements in imaging technologies and therapeutic agents require ongoing updates to clinical guidelines. Collaboration among neurosurgeons, ophthalmologists, and interventional radiologists is essential for optimal CCF management.

## Conclusions

CCFs are a significant health concern, with symptoms like proptosis, ocular redness, and diplopia being key. Early diagnosis and intervention are crucial, especially in cases of significant diplopia. A thorough ocular examination and neuro-ophthalmological assessment are essential. Early identification and management of eyelid swelling and upper lid mass are also crucial. This approach emphasizes the importance of heightened clinical vigilance and timely intervention.
